# Knowledge, Beliefs, and Treatment Practices for Otitis Media in Malawi: A Community-Based Assessment

**DOI:** 10.3390/audiolres15020038

**Published:** 2025-04-06

**Authors:** Enittah Chikuse, Derek Jacobs, Angella Banda, Julia Toman, Jenna Vallario, Danielle Curtis, J. Zachary Porterfield

**Affiliations:** 1 ABC Hearing Clinic and Training Centre, African Bible College, Area 47, Lilongwe, Malawi; chikuse_enittah@abcmalawistudent.net (E.C.); banda_angella@abcmalawistudent.net (A.B.); vallarioj@abcmalawi.net (J.V.); 2 Division of Infectious Disease & International Medicine, Morsani College of Medicine, University of South Florida, Tampa, FL 33620, USA; derekjacobs@usf.edu (D.J.); curtis10@usf.edu (D.C.); 3 Department of Otolaryngology, Morsani College of Medicine, University of South Florida, Tampa, FL 33620, USA; juliatoman@usf.edu

**Keywords:** otitis media, hearing loss, treatment, resource limited, patient understanding

## Abstract

Background: Hearing-related disease is a significant cause of disability worldwide. In resource-limited settings, prevention and early detection are critical for preventing severe disease. Understanding what a population knows and believes with regards to hearing health can be critical in identifying knowledge gaps and developing targeted interventions. Objective: To assess community awareness of hearing health and otitis media (OM) treatment, both modern and traditional, to inform educational programs. Methods: A retrospective review of clinical records from 52 patients (aged 1–79 years) diagnosed with OM during a 3-day hearing health clinic in Kasungu district, Malawi was conducted. Patients diagnosed with OM during the clinic were invited to provide additional details about their hearing health. Surveys contained open-ended questions to assess knowledge and beliefs regarding the cause of their infection and therapies they had previously used for treatment, including home remedies and prescribed medications from allopathic providers or traditional healers. A WHO adapted survey on hearing knowledge was also administered. Results: Hearing loss was identified in 60% of participants. Otoscopy revealed either bilateral or unilateral drainage in 69% of participants and perforation in 73%. Confidence in understanding the causes and treatments of OM was voiced by 60% of participants and 54% had used home remedies as treatment. Of the 11 home remedies used, none aligned with modern medical practice, and only two were recommended by local herbalists. Conclusions: Hearing-related disease contributes significantly to global disability, particularly in resource-limited settings. Educational campaigns to improve hearing health knowledge offer low-cost yet impactful solutions and implementation via partnerships with community leaders and traditional healers can be critical to addressing hearing health challenges. The use of nonantibiotic antimicrobials should be explored further, as these are low-cost and readily available. However, therapeutic alliance between patients and healthcare providers remains crucial.

## 1. Introduction

Hearing-related diseases are a significant cause of disability and morbidity across the globe, as recognized in the World Health Organization (WHO)’s World Report on Hearing [1]. The prevalence of hearing loss continues to rise, with an estimated 2.5 billion people expected to experience some degree of hearing loss by 2050 [1]. However, half of all hearing loss can be prevented by public health measures, and among children, 60% of hearing loss results from preventable causes [2]. Infection, specifically, remains a major preventable cause of hearing loss, with its incidence varying widely by region. The WHO estimates that 98.7 million people have experienced hearing loss due to suppurative otitis media, an infection of the middle ear [1]. Unfortunately, acute otitis media (AOM) and chronic suppurative otitis media (CSOM) disproportionally affect low- and middle-income countries (LMICs) compared with high-income countries (HIC), where the sequelae remain rare [3]. This places the greatest burden on the health systems least equipped to address these challenges.

Malawi, a country in southeastern Africa, exemplifies the challenges LMICs face in addressing preventable hearing loss. However, data on the prevalence of hearing loss and chronic ear disease in Malawi remain limited. A study conducted by Mulwafu found that 11.5% of children aged four to six had bilateral hearing loss, yet only 30% of these children were enrolled in school [4]. Similarly, Hunt et al. found that 12.5% of children had bilateral hearing loss, and nearly a quarter of children aged four to six had hearing loss in at least one ear [5]. More recently, Mtamo et al. found that 68% of patients presenting to an audiology clinic had a pathology consistent with conductive hearing loss [6]. While these studies were limited by sample size and a full description of the hearing loss, they point to a significant burden in a country with scarce hearing healthcare resources.

In resource-limited settings, prevention and early detection are essential to reducing the burden of severe hearing-related diseases. Identifying and addressing gaps in public knowledge about hearing health is a critical step in this process. Prior research among Malawian school teachers demonstrated that targeted educational interventions can significantly improve awareness of hearing health [7]. However, the broader population’s understanding of their hearing health and traditional therapies remains unexplored.

The goal of this study was to assess the knowledge and perceptions of hearing health and traditional hearing therapies in Malawi. A comprehensive assessment of these factors could inform the development of impactful educational campaigns aimed at reducing preventable disease, morbidity, and mortality. Furthermore, insights from this study could serve as a model for enhancing hearing health care strategies in resource-limited settings worldwide.

## 2. Materials and Methods

*Study Design*: A retrospective review was conducted of data collected from patients treated during a hearing health outreach in May 2024 as a collaboration between the African Bible College (ABC) audiology program, Entheos (an American audiology cooperative), Hearing The Call (nonprofit), Kismet: Empowering Health Access (nonprofit), ENT clinicians from the Kasungu District Hospital, and an outreach team from the University of South Florida (USF). The outreach clinic took place over three days at three separate sites in the Kasungu district of Malawi. In the months leading up to the outreach, the ABC team advertised the dates and locations to the local population in coordination with the local ear, nose, and throat (ENT) clinicians at the Kasungu District Hospital. At the clinic, multiple stations were set up that offered a variety of services including otoscopic examinations, wax removal, otoacoustic emission screening, tympanometry, field hearing testing, custom hearing aid molding, hearing aid fitting, and ENT examinations as needed based on clinical presentation of the patient. This outreach clinic facilitated convenience sampling of the population for study enrollment.

As part of the ABC team’s effort to improve the quality of outreach, patients with evidence of infection (purulence, drainage, wet perforations, evidence of fungal infection, or any other evidence of acute or chronic otitis media or otitis externa) were invited to provide additional details about their hearing health. Those who met these criteria and verbally consented to participating were included in the study. No explicit exclusion criteria were defined. The patient or responsible guardian was asked a series of five open-ended questions related to hearing health and ear infections with the goal of assessing the patient’s understanding of what causes ear infections, what treatments are available, and if the available treatments are effective. Additionally, patients were asked a series of ten ‘yes’ or ‘no’ questions adapted from a WHO survey designed to assess hearing health knowledge [7,8]. The survey was administered in Chichewa, the patient’s native language. Although it was not formally validated, author E.C., a native speaker of both languages, translated the survey from English to Chichewa and pretested it on a small group of control subjects to ensure accuracy and readability of the questions. Patient responses were recorded in Chichewa and back-translated into English by author E.C for analysis. These data were collected for quality improvement for clinical outreach and used by the clinic to improve care. This data set was subsequently reviewed for this publication.

While the study was conducted as a quality improvement project, a USF Institutional Review Board (IRB) exemption was obtained for this data review under the IRB number STUDY007654: ABC HCTC Hearing Health Education. Additionally, the USF IRB waived the requirement for consent for this data review.

### 2.1. Quantitative Analysis

Patient charts were reviewed for demographic information including gender, age, education level, occupation, mode of transportation, and travel time to the clinic. Additional information was collected regarding the presence of hearing loss (unilateral vs. bilateral), ear pain, ringing in the ears, risk factors for hearing loss, status of hearing device wear, and presence of drainage or perforation during otoscopic inspection at the outreach clinic. Pure tone audiometry results were also reviewed.

Basic descriptive statistics were used to characterize the data and define means. Statistical analysis was performed using GraphPad Prism version 10.2.3 for Mac, GraphPad Software, San Diego, CA, USA, www.graphpad.com (accessed on 21 April 2024).

### 2.2. Qualitative Analysis

Qualitative analysis of the open-ended responses was performed by reviewing the patients answers individually and discussing key themes that emerged, as a group. The three major themes used were “Knowledge of Causes and Treatments for Ear Infections”, “Knowledge of Prevention and Impact on Livelihood”, and “Treatment Action”. Answers were then coded manually for inclusion in these three thematic areas and subthemes were identified within each area. Patients’ responses were further coded and tabulated as to their certainty regarding their understanding of a particular theme and whether they took action for their infection.

### 2.3. Traditional Healing Methods

Traditional healing methods are common in these communities, so specific attention was paid to assessing which methods were utilized by individuals. The different preparations used by individuals were reviewed, and local traditional herbalists were then queried to better understand home remedies. Information regarding the herb, the part used, the means of usage, and whether it was thought to be useful by the herbalist was recorded.

## 3. Results

### 3.1. Descriptive Analysis

A total of 52 individuals were identified as completing the additional questions regarding hearing health. The mean age of the individuals was 30.2 (SD = 18.7). Of the participants, 27 (51.9%) identified as male, 24 (46.2%) identified as female, and 1 (1.9%) individual did not record a response. Most participants completed their education at the primary level (51.9%) and reported working as a farmer (38.4%). To attend the clinic, an almost equal number of individuals reported taking a bus (26.9%) or riding a bike (25%). It was found that the most common mode of transportation was walking, with 18 individuals (34.6%), and the least common mode of transportation was traveling via car/motorcycle, with only 7 individuals (13.5%). Most individuals traveled between 1 and 2 h (40.4%) or 1 and 30 min (38.5%) to attend the clinic (Table 1).

**Figure 1:**
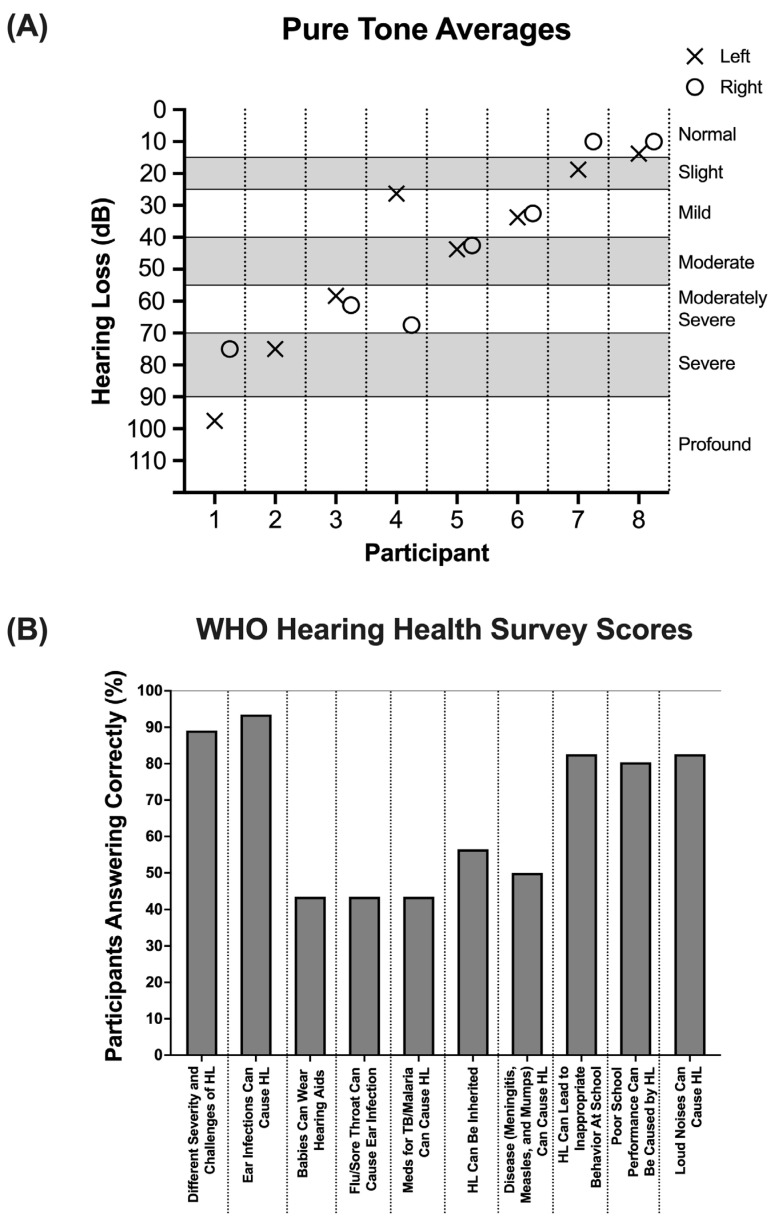
(**A**) Participants’ pure tone average scores in their left and right ears. “X” indicates left ear, and “O” indicates right ear. The severity of hearing loss is indicated by the dB score on the Y-axis. (**B**) The percentage of participants who answered the corresponding WHO Hearing Health Survey question correctly.

Information regarding several otologic variables was ascertained from participants. Over half of the participants reported ear pain (55.8%) and hearing loss (61.5%) bilaterally. Unilateral ear pain, in either the right or left ear, was only reported by five individuals (9.6%), and hearing loss in the right or left ear was reported by only six individuals (11.5%) and four individuals (7.7%), respectively. Despite about 80% of participants reporting either unilateral or bilateral hearing loss, only five individuals had previously worn (5.8%) or were currently wearing (3.8%) a hearing device. There were several risk factors for hearing loss, including noise exposure, family history, and disease incidence. Within this cohort, malaria was the risk factor most reported by individuals (71.2%), followed by family history (30.8%), noise exposure (17.3%), and HIV (11.5%). As part of the outreach, participants underwent otoscopic inspection. Of the 52 participants, ear drainage was reported in 44.2% unilaterally and 25% bilaterally, and perforation was reported in 26.9% unilaterally and 46.1% bilaterally (Table 2). For the eight individuals who also underwent pure tone audiometry, five out of eight had moderate hearing loss or worse, and two had severe to profound hearing loss (Figure 1A). Audiometry results for each of these individuals assigned a pure tone average are presented in Supplemental Figure S1.

### 3.2. Hearing Health Knowledge Analysis: Quantitative Assessment

A total of 46 individuals completed a hearing health knowledge survey containing 10 “yes” or “no” questions adapted from a WHO survey as a part of their participation in the outreach clinic. Notably, several questions were answered incorrectly more frequently than others (Figure 1B). While there was a generally high level of knowledge of certain topics related to hearing health—such as its varying severities and associated challenges (89.1%) and its potential impact on school performance—critical misconceptions were identified. Only 43.5% of individuals correctly answered questions about a baby’s ability to wear a hearing aid, whether the flu or a sore throat could cause ear infections, and whether TB and/or malaria medications could cause hearing loss. Moreover, only 50% of individuals correctly acknowledged that diseases such as meningitis, measles, and mumps could cause hearing loss, and only 56.5% of individuals correctly acknowledged that hearing loss could be inherited.

### 3.3. Hearing Health Knowledge Analysis: Qualitative Assessment

**Figure 2:**
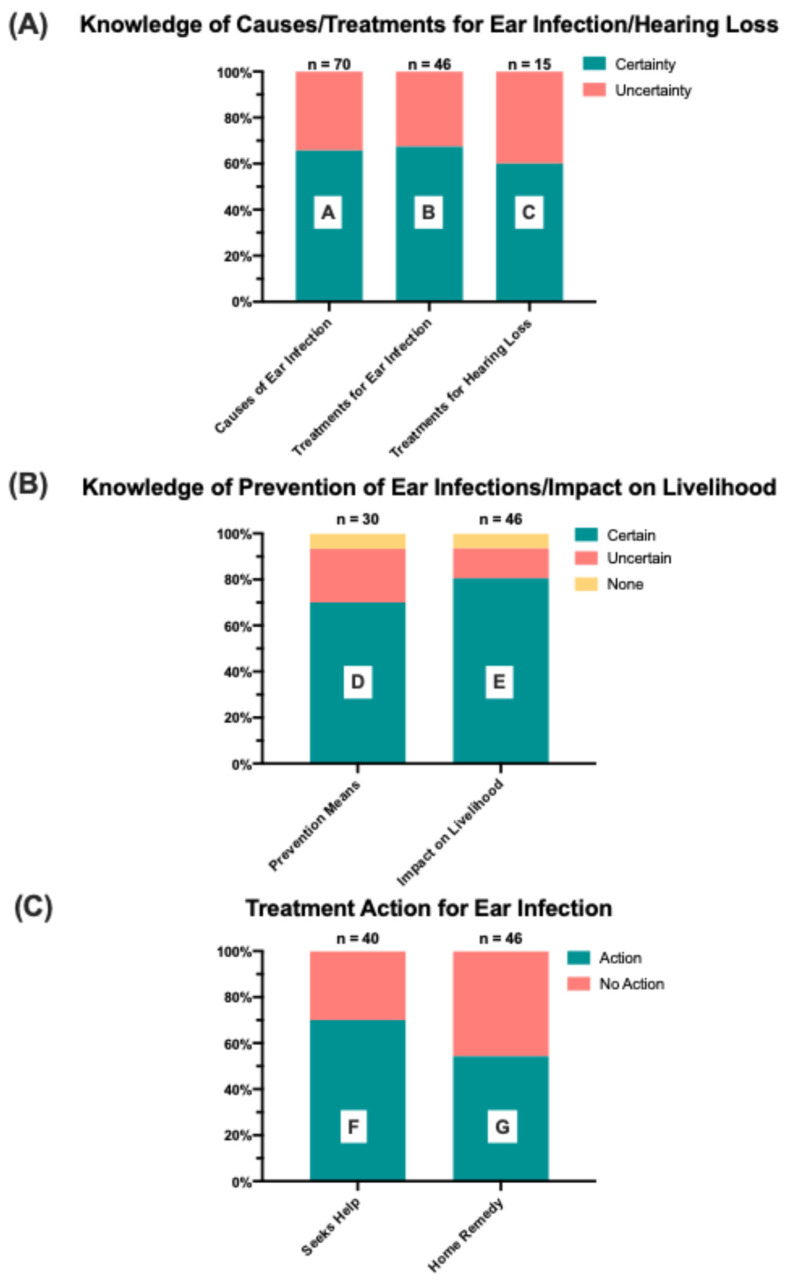
Thematic coding of open-ended responses to assess knowledge and actions related to ear infections. Response sample sizes are annotated above each bar; “A”, “B”, “C”, “D”, “E”, “F,” and “G” refer to quotes in Table 2. (**A**) Responses on knowledge about the causes for ear infection and treatments for ear infection and hearing loss, coded for certainty (green) and uncertainty (pink). (**B**) Responses on knowledge about means of preventing ear infection and the impacts on livelihood, coded for indications of certainty (green), uncertainty (pink), or none (yellow). (**C**) Responses assessing whether patients sought outside help or used home remedies for treatment, coded for indications of took action (green) or no action (pink).

Participants completed a quality improvement interview containing five open-ended questions related to hearing health and ear infections to assess their understanding of ear infection root causes, treatment availability, and effectiveness. Thematic analysis of participant responses revealed three key themes: “Knowledge of Causes and Treatments for Ear Infections”, “Knowledge of Prevention and Impact on Livelihood”, and “Treatment Action”. These overarching themes were broken down into several subthemes and responses were coded according to the legends in Figure 2. Many participants provided responses that exhibited overlapping themes. However, each mention of a subtheme was counted as a separate instance, with the sample size of subtheme instances denoted over the corresponding subtheme bar graph.

Within the theme of “Knowledge of Causes and Treatments for Ear Infections”, three subthemes emerged: “Causes of Ear Infections”, “Treatments for Ear Infections”, and “Treatments for Hearing Loss” (Figure 2A). Responses were coded according to whether they indicated participant certainty or uncertainty regarding knowledge pertaining to each subtheme. For the subtheme “Causes of Ear Infections”, a coding of certainty illustrated that the participant was certain about their cause or other causes of infection, whereas uncertain illustrated uncertainty of their cause or other causes of infection. For the subthemes “Treatment of Ear Infection” and “Treatment of Hearing Loss”, a coding of certainty illustrated the participant was certain about possible treatments regardless of whether they deemed it effective or not, whereas uncertain illustrated uncertainty of any forms of treatment. Across all three subthemes, over 60% of responses for each subtheme indicated certainty regarding knowledge of causes of ear infections and treatment of ear infections and hearing loss. Examples of these responses are found in Table 3 in rows A, B, and C of individual responses pertaining to causes and treatments.

Within the “Knowledge of Prevention and Impact” theme, the subthemes of “Prevention Means” and “Impact on Livelihood” emerged (Figure 2B). Responses were coded according to whether the individual indicated that they believed a means of prevention or potential impact existed and whether they indicated certainty or uncertainty regarding knowledge pertaining to each subtheme. Only 6.7% and 6.5% of interview responses indicated that individuals believed that there was no possible means of prevention and no impact on livelihood due to ear infection, respectively. Most responses indicated certainty regarding the knowledge of prevention means (70%) and ear infection’s impact on livelihood (80.5%). Rows D and E in Table 3 illustrate examples of patient responses on the effects on livelihood or prevention means.

“Treatment Action” was the third major theme identified, and during further analysis, the subthemes of “Seeking Help” and “Home Remedies” emerged (Figure 2C). Responses were coded for each subtheme according to if they indicated that the individual took some sort of action, whether it was seeking help or home remedies, or that they did not take action to address infection. Amongst the responses that aligned with the subtheme of “Seeking Help”, 70% indicated that individuals sought some form of outside assistance for their ear infections. Exemplified by the quotes in row F of Table 3, these outside sources included traditional healers and clinicians, with only 30% of responses indicating they sought no outside help. Amongst the responses that aligned with the subtheme of “Home Remedies”, almost equal proportions of responses occurred indicating either action or no action, with 54.3% of responses within that subtheme indicating action with home remedies and 45.7% indicating no action with home remedies. As outlined in row G of Table 3, these home remedies included using herbs, cloth, matchsticks, chicken feathers, urine, and soot.

### 3.4. Traditional Remedies

Participants identified 11 herbs utilized as home remedies to treat CSOM, which included madzi a anyezi (onion water), mathulisa, cham’mwamba (moringa), nthethanyerere, masamba a nandolo (pea leaves), madzi a mtengo wa nthochi (banana stem water), aloe vera, masamba a tomato (tomato leaves), chamba (marijuana), and likhodza (Table 4). In addition to these herbs, some also reported using salt, soot (mwaye or mwawi), urine, and a methylated spirit. The herbs were used topically rather than via systemic/oral ingestion. Of the herbs used by participants, only onion water and aloe vera were confirmed via interviews with the local herbalists to be recommended as effective for treating CSOM, because of their antimicrobial and antifungal properties. The remainder of the remedies were considered ototoxic by the herbalists.

Interviews conducted with two local herbalists revealed additional recommended herbal treatments for CSOM, including onion water, aloe vera, neem oil, and water from mustard leaves. Of these recommended treatments, only two were used by individuals attending the outreach clinic (onion water and aloe vera), whereas nine of the herbs referenced by clinic participants were not recommended by the local herbalists. The route of use varied between the herbalists, with some recommending only topical use while others recommended topical use and ingestion. All herbalists recommended consultation with a traditional healer for these remedies to ensure the correct dosage, proper preparation, and selection of the herb. All noted that the lack of consultation was likely to result in worsened disease and affected hearing.

## 4. Discussion

This is the first review that has sought to directly assess the level of hearing awareness among patients with acute or chronic ear disease who received care through a hearing outreach clinic. The extent of ear disease in this cohort was remarkable, with 61.5% reporting bilateral hearing loss yet only 3.8% reporting they were currently using a hearing device. Otoscopic findings highlighted the clinical burden of ear disease, with almost half of individuals noted to have bilateral perforations (46%) and at least one draining ear (44%). Although this cohort was referred for further evaluation from a larger hearing screening effort, leading to some expected enrichment for pathology, the burden of undertreated disease remained significant. These findings align with findings from the African Bible College (ABC) clinic in Lilongwe, Malawi, where 83% of patients presented with outer or middle ear pathology, and 68% presented with pathologies often associated with conductive hearing loss [6].

Barriers to hearing healthcare were evident, as 40% of individuals reported traveling for 1–2 h for outreach care. Additionally, nearly two-thirds of patients arrived at the clinic on foot or by bicycle, further emphasizing the challenges to accessing hearing healthcare. These travel demands, coupled with the severe shortage of ear care specialists, further limit access to timely diagnosis and treatment. As of 2017, only two ear, nose, and throat (ENT) surgeons and three audiologists served a population of approximately 17.2 million [16]. Although the number of trained audiologists has increased since 2021, because of the graduation of Malawi’s first ten audiologists with Bachelor of Science degrees in Audiology by ABC, job opportunities and retainment of trainees remain low. Despite significant barriers to care, 70% of patients in our outreach had sought treatment for their infection, which was higher than expected, as ear discharge is often considered normal and dismissed without formal medical attention [17,18].

Beyond provider shortages and geographic barriers, gaps in hearing health awareness also contribute to delayed diagnosis and treatment. While most participants (93.5%) acknowledged that hearing loss affected livelihood and was preventable (93.3%), the hearing health knowledge survey revealed key knowledge gaps. Many were unsure about whether babies could wear hearing aids, whether flu and/or a sore throat could cause ear infections, and whether TB and/or malaria medications could cause hearing loss. More than half (56.5%) answered these questions incorrectly, mirroring previous findings from Malawi, where 76%, 44%, and 41%, respectively, answered these questions incorrectly [7]. These findings highlight the need for targeted educational interventions to correct misconceptions and improve hearing health literacy. Limited awareness of these hearing loss risk factors is particularly concerning in a population with high malaria prevenance (a remarkable 71% in this cohort). Additionally, a lack of knowledge about the connection between infectious diseases and ear pathology is concerning, as neglecting these illnesses, especially in children, can lead to lifelong otologic disease [19].

Limited awareness of hearing health risks is not unique to this population and has been observed in other global settings. Although research regarding hearing health knowledge and awareness in the specific context of Malawi is scarce, additional studies in countries with high levels of AOM and CSOM have begun to identify hearing health knowledge trends. For example, a study in rural south India found that 50% of participants showed deficits in knowledge regarding otitis media risk factors, while a Nigerian study reported that knowledge of otitis media risk factors varied by socioeconomic status, thus highlighting the need to integrate healthcare education with economic development programs [17,20]. Similarly, a study conducted in the Netherlands found an increased risk of CSOM (OR 14.1) among children whose parents had lower education levels [21]. Regarding knowledge about providers and treatment, a study in rural South Africa found that only 14% of participants were aware of the audiology profession, and only 5% had previously seen an audiologist [22]. In contrast, a study in rural south India found that earaches were often treated with traditional or home remedies (67.2%) or were not treated at all (26.4%) [17]. Continued research is essential to fully assessing current understanding and treatment practices in developing regions.

To the authors’ knowledge, this is the first study to specifically collect data on traditional remedies used for otitis media by patients and to evaluate their use with local traditional healers. Interviews with herbalists provided additional insight into the recommendations patients receive when seeking medical advice from different sources. Notably, many of the treatments used by patients were not recommended by the traditional healers, who based their recommendations on the belief that specific herbs have natural antimicrobial and/or antifungal properties for CSOM. Although there is very limited evidence that supports the antimicrobial properties of these specific herbs, interest in nonantibiotic and antimicrobial treatments for CSOM has been growing. For example, a 2012 study in Cape Town, South Africa found that boric acid was as effective as quinolone drops, the current standard of care, but significantly more affordable [23]. Additionally, it was also found that Quadriderm cream was effective in 85% of patients who failed first-line therapy, with none of the treatments showing ototoxic effects [23]. In 2020, Van Straten et al. examined the utility of antiseptic agents against common organisms of CSOM in vitro. They found that boric acid powder and 5% povidone iodine showed promising results, with 3.25% aluminum acetate producing excellent activity against Pseudomonas aeruginosa [24]. Another commonly used antimicrobial for CSOM is acetic acid, though its effectiveness varies widely, likely because of differences in disease presentation and patient adherence [24,25,26].

This study’s findings, coupled with findings from the literature, suggest that targeted educational interventions may serve as a cost-effective opportunity to enhance hearing health knowledge, leading to earlier identification, intervention, and improved outcomes. Specifically, given real resource limitations in developing regions, an improved understanding of the various antimicrobial properties of readily available natural agents has the opportunity to improve access to care for patients. However, comprehensive evaluations of the ototoxic potential of these herbs remains scarce. A systematic review conducted in 2017 was able to identify only seven randomized trials evaluating herbal medicines for acute otitis media treatment, all of which were identified to be poor-quality trials [27]. Further research into the potential ototoxicity of herbal otitis media treatments and improved education surrounding the safety profiles of these various home remedies will be critical to preventing accidental ototoxic events.

*Limitations*:

There are several limitations to this study. Convenience sampling at the outreach clinic increased the risk of selection bias, as patients had self-presented to the outreach initiative and thus were likely to have current or prior experience with ear infections and poor hearing health. As such, these patients may not be representative of the broader population of Malawi. Furthermore, the ability to obtain the additional quality improvement data occurred on a rolling basis within the larger outreach setting, and thus, not all patients with otitis were interviewed. Notably, statistical analysis was limited by the number of patients who were able to be enrolled in the study. Given that the study was not designed to identify differences in a primary outcome but rather to describe the landscape of beliefs and treatment patterns related to otitis media in Malawi, we elected to present the data as a qualitative report. Follow-up studies to more completely define these trends and compare differences between key subgroups would be valuable and are underway. Finally, the quality improvement questions were initially conceptualized in English, but the majority of patients primarily spoke Chichewa, and there was an occasional need for rephrasing or prompting, which may have introduced some variance in response.

## 5. Conclusions

Assessing a patient’s understanding of their disease process is critical to treatment. An understanding of a community’s beliefs and practices allows for the development and implementation of targeted interventions to improve early detection and treatment of disease. Here, we identified specific gaps in knowledge about the cause, impact, and treatment of otitis media in a patient population heavily burdened by this disease. Notably, many of these patients had attempted home remedies, which both our clinical staff and the herbalists interviewed for this manuscript felt were of limited utility and potentially harmful. However, given the benefit of effective nonantibiotic treatment for otitis media, additional evaluation of these options, particularly in the context of evaluating therapies such as boric acid and acetic acid, is valuable.

Optimal care is achieved by aligning patient needs and understanding with the best available evidence. However, this depends on the patient’s underlying understanding of their disease, the availability of treatment, and a strong therapeutic alliance with their healthcare provider. Although this study was conducted in a rural Malawian setting, we believe these findings have a broad applicability across a wide range of healthcare contexts.

## Figures and Tables

**Table 1:** Distribution of demographic variables.

		N = 52 ^1^
**Age**		30.2 (18.7)
**Gender**	**Male**	27 (51.9%)
	**Female**	24 (46.2%)
	**Unknown**	1 (1.9%)
**Education Level**	**Primary**	27 (51.9%)
	**Secondary**	7 (13.5%)
	**None**	3 (5.8%)
	**Other**	10 (19.2%)
	**Unknown**	5 (9.6%)
**Occupation**	**None**	5 (9.6%)
	**Student**	8 (15.4%)
	**Farmer**	20 (38.4%)
	**Businessman**	4 (7.8%)
	**Other**	7 (13.5%)
	**Unknown**	8 (15.4%)
**Mode of Transportation to Clinic**	**Walk**	18 (34.6%)
	**Bus**	14 (26.9%)
	**Bicycle**	13 (25%)
	**Car/Motorcycle**	7 (13.5%)
**Travel Time to Clinic**	**1 to 30 min**	20 (38.5%)
	**30 min to 1 h**	8 (15.4%)
	**1 to 2 h**	21 (40.4%)
	**2 to 4 h**	3 (5.8%)

^1^ n (%); Mean (SD).

**Table 2:** Distribution of otologic variables.

		N = 52 ^1^
**Ever Worn a Hearing Device**	**Currently Worn**	2 (3.8%)
	**Previously Worn**	3 (5.8%)
**Ear Pain**	**Right**	5 (9.6%)
	**Left**	5 (9.6%)
	**Both**	29 (55.8%)
**Ringing In Ears**	**Right**	9 (17.3%)
	**Left**	3 (5.8%)
	**Both**	14 (26.9%)
**Risk Factors for Hearing Loss**	**Family History**	16 (30.8%)
	**Noise Exposure**	9 (17.3%)
	**Meningitis**	2 (3.8%)
	**Tuberculosis**	1 (1.9%)
	**HIV**	6 (11.5%)
	**Malaria**	37 (71.2%)
**Hearing Loss**	**Right**	6 (11.5%)
	**Left**	4 (7.7%)
	**Both**	32 (61.5%)
**Otoscopic Inspection**	**Unilateral Drainage**	23 (44.2%)
	**Bilateral Drainage**	13 (25%)
	**Unilateral Perforation**	14 (26.9%)
	**Bilateral Perforation**	24 (46.1%)

^1^ n (%).

**Table 3:** Example quotes corresponding to qualitative subthemes in Figure 2.

**Subtheme I:** Causes of Ear Infection “A”	“I do not know what caused my ear infection. Other cause is poor hygiene”. “I just realized it has started, but growing up I was mostly beaten on the ears. Other causes I do not know”. “Ear infection started as a result of suffering from malaria. And other causes are cough and discharge”. “It was caused by working as a farmer. Other causes are noise exposure”.
**Subtheme II:** Treatment for Ear Infection “B”	“Medications, suctioning, and operation. These treatments work”. “Cooking oil; does not hel”. “Ear drops, suctioning and dry mopping. Yes, they help”. “Ear drops, prayers. They do not help since there has been no improvemen”.
**Subtheme III:** Treatment for Hearing Loss “C”	“No treatment for deafnes”. “Hearing aids and they work”. “Using medications, suctioning, operation, hearing aids. Yes, they work”. “Uses herbs and medicines from hospital. They do not work.
**Subtheme IV:** Means of Prevention “D”	“Observing good ear hygiene, and rushing to the hospital “Avoiding loud noises and dust in the ear”. “Not letting water into the ear and not poking the ear”. “Not bathing in dirty water”.
**Subtheme V:** Impact on Livelihood “E”	“Missing some things when people are talking, being isolated and discriminated”. “No problems can arise from ear infection. Ear infections cannot cause hearing los”. “Being mocked, isolated, having no friends and being a victim of ill speaking. I do not know, but I think it can cause hearing loss”. “Dropping out of school. Trouble hearing. Yes, it can cause hearing loss”.
**Subtheme VI:** Sought Help “F”	“I have seen traditional healers for help”. “I consult no one for help”. “I see clinicians and traditional healers”. “I do go to the hospital and meet the doctor in charge or assistant”.
**Subtheme VI:** Home Remedies “G”	“I have used herbs before, but I do not know them, and I use a piece of cloth”. “I was using urine, soot (Mwaye), herbs (luni), penicillin and other medications from the hospital”. “Taking medications; applying onion water and salt; and cleaning using matchstick”. “I use chicken feather to remove the pus”.

**Table 4:** Common medicinal herbs mentioned by participants.

**Luni Leaves**	**Description**	An edible vegetable reported to be bitter.
	**Preparation**	Grind the leaves and filter the fluid through a clean cloth.
	**Use**	Apply drops of the filtered fluid filtered in the ear.
Scales of Onion Bulb **	**Description**	An edible vegetable used in cooking and for medicinal purposes [9].
	**Preparation**	Remove the roots, crush the onion bulbs, and sieve out the fluid.
	**Use**	Apply drops of the fluid into the ear.
**Mathulisa Leaves**	**Description**	Plant/weed found on local farms. Used for medicinal purposes.
	**Preparation**	Grind the leaves and extract the juice from the leaves using a cloth.
	**Use**	Apply drops of filtered fluid in the ear or drink boiled water.
**Moringa Leaves**	**Description**	Plant/vegetable used for various health problems (i.e., heart disease, blood pressure, diabetes) [10].
	**Preparation**	Grind the fresh leaves and squeeze out the fluid using a cloth.
	**Use**	Apply drops of the filtered fluid in the ear.
**Nthethan-yerere Roots**	**Description**	A plant mostly found on farms and mountains. Considered a weed.
	**Preparation**	Mix the roots with hot water and soak for 1 to 2 days.
	**Use**	Apply drops of the fluid in the ear.
**Banana Trunk**	**Description**	Fruit is used for food and medicine. Leaves are used as a wrapper to store food. Stem is used for food, detoxification, and medicine [11].
	**Preparation**	Break and squeeze the banana trunk to extract the fluid.
	**Use**	Apply drops of the fluid in the ear.
Aloe Vera Gel **	**Description**	Used for cosmetics and medicinal purposes (i.e., wounds, burns, dysentery, inflammation) [12].
	**Preparation**	Remove the spines, cut the leaf open, and remove the gel.
	**Use**	Apply a small portion of the extracted gel in the ear.
**Tomato Leaves**	**Description**	Fruit plant used when cooking to flavour food [13].
	**Preparation**	Pluck tomato leaves and clean them. Crush them to extract the juice.
	**Use**	Apply drops of the filtered juice in the ear.
**Marijuana (Cannabis)** **Seedling Leaves**	**Description**	Used for fibre, row, food, and medicine. Also used religiously and recreationally [14].
	**Preparation**	Soak dry leaves in warm water for a day, sieve. Soak or crush seeds and add them to water, sieve. Both fresh and dry leaves can be used.
	**Use**	Apply the water to the ear, but do not put the seeds in the ear.
**Likhodza Leaves**	**Description**	Considered a weed but used as medicine for problems such as cancer.
	**Preparation**	Dethorn leaves, smash, add to water. Use cloth to squeeze fluid out.
	**Use**	Apply drops of the filtered fluid in the ear.
**Pigeon Pea Leaves**	**Description**	A perennial legume. Used raw or cooked [15].
	**Preparation**	Grind the fresh leaves, place them on a cloth, squeeze out fluid.
	**Use**	Apply drops of the filtered fluid in the ear.

** = recommended.

## Data Availability

The data presented in this study are available on request from the corresponding author because of subject privacy reasons.

## Supplementary Materials

The following supporting information can be downloaded at: https://www.mdpi.com/article/10.3390/audiolres15020038/s1, Figure S1: Audiometry results.

## Abbreviations

The following abbreviations are used in this manuscript:

OM	Otitis media
WHO	World Health Organization
AOM	Acute otitis media
LMICs	Low- and middle-income countries
HIC	High-income countries
ABC	African Bible College
ENT	Ears, nose, and throat
HIV	Human immunodeficiency virus
TB	Tuberculosis
CSOM	Chronic suppurative otitis media
