# Supplementary material for: Knowledge, Beliefs, and Treatment Practices for Otitis Media in Malawi: A Community-Based Assessment
Source: Audiol Res. 2025 Apr 6;15(2):38. doi: 10.3390/audiolres15020038 (PMC12024340; doi:10.3390/audiolres15020038)
Supplement: Supplementary file 1 [file audiolres-15-00038-s001.zip › Supp Figure Caption.pdf]

SUPP FIG 1: Audiometry results for eight different participants. “X” indicates left ear and “O” indicates right ear. The severity of hearing loss is indicated by the dB score on the Y-axis at a given frequency indicated by the Hz on the x-axis.
